# Statins and Peripheral Arterial Disease: A Narrative Review

**DOI:** 10.3389/fcvm.2021.777016

**Published:** 2021-11-22

**Authors:** Sergio Jansen-Chaparro, María D. López-Carmona, Lidia Cobos-Palacios, Jaime Sanz-Cánovas, M. Rosa Bernal-López, Ricardo Gómez-Huelgas

**Affiliations:** ^1^Internal Medicine Service, Regional University Hospital of Malaga, Instituto de Investigación Biomédica de Málaga (IBIMA), University of Malaga (UMA), Malaga, Spain; ^2^CIBER, Fisiopatología de Obesidad y Nutrición (CIBERobn), Instituto de Salud Carlos III, Madrid, Spain

**Keywords:** statins, peripheral arterial disease, cardiovascular risk, amputation, critical limb ischemia

## Abstract

Peripheral arterial disease (PAD) is a highly prevalent atherosclerotic condition. In patients with PAD, the presence of intermittent claudication leads to a deterioration in quality of life. In addition, even in asymptomatic cases, patients with PAD are at high risk of cardiac or cerebrovascular events. Treatment of PAD is based on lifestyle modifications; regular exercise; smoking cessation; and control of cardiovascular risk factors, including hypercholesterolemia. A growing number of studies have shown that statins reduce cardiovascular risk and improve symptoms associated with PAD. Current guidelines recommend the use of statins in all patients with PAD in order to decrease cardiovascular events and mortality. However, the prescribing of statins in patients with PAD is lower than in those with coronary heart disease. This review provides relevant information from the literature that supports the use of statins in patients with PAD and shows their potential benefit in decreasing lower limb complications as well as cardiovascular morbidity and mortality.

## Introduction

The European Society of Cardiology uses the term peripheral arterial diseases to describe atherosclerotic involvement of all arterial territories except the aorta and coronary arteries (1). Although this concept encompasses the involvement of numerous arteries, in practice and for this article, the term peripheral arterial disease (PAD) refers to stenosis of the vessels of the lower extremities of atherosclerotic origin, excluding occlusion by other processes such as embolisms, fibromuscular dysplasia, or vasculitis.

PAD has a high prevalence. It is estimated that there are more than 200 million people with PAD in the world (2, 3). The prevalence of PAD, defined as the presence of an ankle-brachial index (ABI) <0.9, is 4.3% in subjects ≥40 years, but increases to 14.5% in subjects ≥70 years (4). The human and economic cost of this disease is very high. Given the relationship of PAD to age (5, 6), an increase in its prevalence and the costs it generates is expected due to increased survival and progressive population aging (2, 7, 8).

The clinical manifestations of PAD are variable. It can be asymptomatic or manifest as intermittent claudication, a clinical condition characterized by pain in the legs during exercise due to insufficient oxygen supply to the muscles that improves with rest. In more severe cases, acute limb ischemia or critical limb ischemia (CLI) may occur. Acute limb ischemia is defined as acute (<2 weeks), severe hypoperfusion of the limb characterized by pain, pallor, pulselessness, poikilothermia, paresthesias, and paralysis (9). CLI is characterized by chronic (≥2 weeks) ischemic rest pain, non-healing wound/ulcers, or gangrene in one or both legs attributable to objectively proven arterial occlusive disease (9). The onset of symptoms has a significant negative impact on patients' quality of life due to the functional limitations derived from intermittent claudication and amputation of lower extremities in advanced cases.

Patients with PAD, including asymptomatic patients, have an increased risk of local ischemic events due to atherothrombotic complications in the vessels of the legs, as well as systemic complications (myocardial infarction and stroke) and increased mortality (10–14). The relative risks are two to three times higher than that of age- and sex-matched subjects without PAD (10). Cardiovascular and all-cause mortality after a diagnosis of PAD is higher than after myocardial infarction (15–17). Mortality from PAD increased between 1990 and 2013, despite the fact that mortality decreased for all cardiovascular diseases (18). More recent data from the United Kingdom show that between 2006 and 2015, the incidence of coronary heart disease remained stable and its cardiovascular mortality rate decreased whereas incidence of PAD decreased, yet its mortality remained significantly unchanged (19).

In patients with PAD, the risk of progression of lower limb involvement is lower than that of suffering a cardiovascular event in another territory (20, 21). In 5 years of follow-up on patients with *de novo* intermittent claudication, 80% remained stable; ~1% required amputation; 37% had a major cardiovascular event; and 27% died, 54% of which were due to cardiovascular causes (21). However, other studies have found a higher rate of disease progression and lower extremity complications. In a meta-analysis that included 35 articles, patients with symptomatic PAD had 5-year cumulative cardiovascular mortality that was higher than that of the reference population (13 vs. 5%). In this follow-up period, 7% of asymptomatic patients went on to have intermittent claudication and 21% of patients with intermittent claudication developed CLI, with 4–27% requiring amputation (22).

Patients with PAD often have several associated cardiovascular risk factors (4) and frequently have other diseases that entail increased cardiovascular risk, such as metabolic syndrome (23), hepatic steatosis (24), or chronic kidney failure (25). In addition, atherosclerotic involvement of other vascular territories such as coronary or cerebral territories is very frequent in these patients (26, 27). Therefore, PAD may serve as a marker of the presence of atherosclerosis in other territories or of susceptibility to polyvascular involvement (6). Given the high cardiovascular risk associated with PAD, its diagnosis in any patient, regardless of the presence of symptoms, should be viewed as an opportunity to intensively control their cardiovascular risk factors and prevent future events. From the moment of diagnosis, treatment should be optimized with drugs that have been shown to improve symptoms and functional and vital prognosis, such as statins, antiplatelet agents, and antihypertensive drugs (1).

The treatment of patients with PAD should be aimed on the one hand at improving the disease's symptoms and increasing ambulation ability and on the other hand at decreasing the overall cardiovascular risk. The control of cardiovascular risk factors, especially tobacco cessation, is key in all cases, while revascularization should be reserved for symptomatic patients. Since lowering low-density lipoprotein cholesterol (LDL-C) reduces the risk of lower limb complications and the development of cardiovascular events, lipid-lowering therapy is strongly recommended in patients with PAD in all current guidelines (1, 9, 28, 29). However, the cardiovascular risk associated with PAD has traditionally been underestimated and the control of cardiovascular risk factors in these patients remains deficient compared to subjects who have had a myocardial infarction or stroke (16, 30–36). This could explain why mortality from PAD has changed little in the last three decades (12, 18, 19, 37). Furthermore, there are few trials conducted specifically in patients with PAD; indeed, current recommendations have been adapted from other at-risk populations, particularly from those with ischemic heart disease.

In light of the foregoing, PAD should be considered a marker of generalized atherosclerosis and these patients should be treated as subjects with high cardiovascular risk in which strict targets should be sought for all cardiovascular risk factors, including LDL-C.

## Lipids and Peripheral Arterial Disease

Atherosclerosis is a chronic inflammatory disease of the arteries and constitutes the pathological process underlying most cardiovascular diseases. LDL-C plays a major role in the development of atherosclerotic plaque. In areas of the arterial wall with a dysfunctional endothelium, LDL particles penetrate the arterial intima. The probability of these particles invading the dysfunctional endothelium depends on plasma LDL levels. After crossing the endothelium, LDL is retained and modified after being oxidized or glycosylated. The accumulation of these modified particles triggers an inflammatory response, with the release of a series of molecules (adhesion molecules, reactive oxygen species, and pro-inflammatory cytokines and chemokines) that attract monocytes and lymphocytes to the interior of the vascular wall. Monocytes are transformed into macrophages and, after absorbing oxidized lipids, into foam cells. In addition, smooth muscle cells proliferate and produce interstitial collagen and elastin, resulting in the formation of a fibrous layer that covers a lipid-rich necrotic core, thereby forming the atheroma plaque. These plaques can produce ischemia by occluding the vascular lumen, which compromises blood flow, or by rupturing and causing the formation of thrombi. Both mechanisms can lead to the onset of PAD symptoms when they affect the vessels of the lower limbs.

The main risk factors for the development of PAD are those related to arteriosclerosis, such as age, high blood pressure and hyperlipidemia, and especially tobacco use and diabetes. Compared to coronary heart disease, there are few studies that have looked at the role of plasma lipids as risk factors for the development of PAD.

Total cholesterol and LDL-C are the lipid parameters that have most often been associated with PAD. The Framingham Heart Study has shown that a 40 mg/dL increase in total cholesterol was associated with an increased relative risk of 1.2 [95% confidence interval (CI) 1.1–1.3] of developing intermittent claudication (38). Other studies have confirmed the relationship between elevated total cholesterol levels and PAD (39–42), which in some cases may be even more potent than with coronary heart disease (43). Total cholesterol levels are inversely correlated to ABI values (40) and a relationship has been observed between the time since onset of hypercholesterolemia and incidence of PAD in men (44). In regard to LDL-C, its role as an independent risk factor for the development of PAD has also been observed (45, 46). Patients with lower ABI values have higher LDL-C levels (40) and these levels are an independent predictor of deterioration in the ABI (47). Conversely, other studies have found no relationship between total cholesterol or LDL-C levels and the development of PAD after applying multivariate regression models (48, 49). Although the causal role of LDL-C in the formation of atherosclerotic plaques is indisputable (50), atherosclerosis is a systemic disease and the pathological features of plaques are different depending on the vascular territory affected. While acute coronary syndrome occurs due to the formation of a thrombus after rupture or erosion of an atherosclerotic plaque in the coronary arteries, in most peripheral arteries with significant stenosis, the presence of extensive thrombi has been observed and in many cases is in non-significant atherosclerosis (51). In fact, patients with severe chronic ischemia of the legs have a prothrombotic coagulation profile compared to control patients (52). It is possible that these differences in plaque characteristics may explain the disparity in outcomes in studies that have examined the relationship between LDL-C and PAD.

High-density lipoprotein cholesterol (HDL-C) has a protective effect on the development of atherosclerosis. Numerous studies have consistently shown that low levels of HDL-C are one of the most potent lipid risk factors for the development of PAD (38, 40, 41, 45, 49, 53). The role of triglycerides as a risk factor for PAD seems less clear, since some studies do not find an independent relationship after adjusting for other lipid parameters (38, 40, 42). However, other studies have found a significant, independent relationship between triglycerides and PAD (39, 46, 48, 54, 55). Triglyceride levels have been linked to the progression of PAD (56). In addition, in patients with PAD treated with statins, the presence of elevated triglycerides predicts the need for revascularization, which has led to the suggestion that hypertriglyceridemia may counteract one of the benefits provided by statins: reduced need for limb revascularization (57). A relationship between non-HDL cholesterol, which includes all atherogenic lipoproteins, and PAD incidence has been observed in several studies (42, 58), but has not been confirmed in others (48). The role of the total cholesterol/HDL-C ratio (46, 53) has also been analyzed, and it has proved to be the most potent independent lipid predictor in a study that compared the predictive value of 11 lipid and non-lipid parameters as risk factors for the onset of symptomatic PAD (46). Other lipid factors that have been associated with PAD are elevated levels of apoprotein B (46, 59) and lipoprotein (a) (60–64). As with HDL-C, apolipoprotein A-1 is a protective element and its levels inversely correlate to PAD (46, 48).

A different distribution of atherosclerotic lesions of the lower extremities has been described depending on the risk factors for onset of PA. Hypercholesterolemia is more related to the involvement of large vessels and lesions above the knees (65). It has also been suggested that the lipid profile may predict the angiographic complexity of PAD lesions, given the finding that patients with more complex lesions have higher triglyceride/HDL-C ratio values (66).

## Effects of Statins on Plasma Lipids and Arteriosclerosis

Statins are the most commonly used drug class for reducing plasma cholesterol. Its mechanism of action is the inhibition of the enzyme 3-hydroxy-3-methylglutaryl-coenzyme A reductase, which plays a key role in the hepatic synthesis of cholesterol. By decreasing this synthesis, statins reduce circulating LDL-C, increasing its absorption by the liver. In addition, these drugs also increase hepatic absorption of very low-density lipoproteins, thereby decreasing plasma triglyceride concentrations. They also increase plasma HDL-C; decrease the number of small, dense LDL particles; and may improve HDL functionality.

Numerous studies have shown that statins are capable of reducing cardiovascular morbidity and mortality. At present, they are key drugs in the primary and secondary prevention of cardiovascular disease. Their beneficial effect is independent of the initial levels of LDL-C. However, there are no studies in the literature that exclusively analyze patients with PAD that set specific LDL-C targets for this disease. Indeed, the targets established in the guidelines are based on studies conducted in subjects with coronary heart disease. Despite this, statins are a basic pillar of medical treatment for patients with symptomatic or asymptomatic PAD.

The beneficial role of statins is mainly attributed to their ability to lower LDL-C levels, which slows the progression of atherosclerosis and can even lead to regression of atheroma plaque. However, the positive effects of statins go beyond improving the lipid profile. These drugs have other antiatherogenic and cardioprotective effects mediated by their pleiotropic actions, which contribute globally to reducing cardiovascular events and improving lower extremity functionality. Statins have antioxidant properties; improve endothelial function; have antithrombotic and immunomodulatory actions; inhibit the growth of smooth muscle cells, cell adhesion, and C-reactive protein (CRP) secretion; and reduce inflammation of the vascular wall, contributing to stabilizing atheroma plaques (67–70). They also decrease systemic inflammation (71) and have vasodilator action by improving the endothelial activity of nitric oxide synthase and the release of nitric oxide, which has a vasodilator effect and inhibits platelet aggregation (72, 73). In addition, they inhibit endothelin-1, a potent vasoconstrictor (74).

At the femoral level, high-dose statins decrease intima-media thickness and improve the composition of the plaque or cause plaque regression (75–77). Statins have been shown to improve endothelial function and neovascularization by increasing the number and function of endothelial progenitor cells (78). The authors of the SISOPAD study, which showed an improvement in ABI values in patients with intermittent claudication treated with simvastatin for 1 year, suggest that this drug probably improved endothelial function and reduced femoropopliteal atherosclerosis (79). In addition, studies in mice suggest that statins have the potential for therapeutic angiogenesis through the improvement of blood perfusion in the extremities of animals experiencing acute ischemia through mechanisms independent of their lipid-lowering actions (80, 81).

## Effects of Statins on Intermittent Claudication and Ambulation Ability

Intermittent claudication is the most frequent clinical manifestation of PAD. It entails a functional limitation and a decrease in the quality of life of patients who present with it. One of the goals of treating patients with PAD is to improve their symptoms and prevent atherothrombotic complications of the extremities.

Statins prevent the development of symptomatic PAD in high-risk patients. In a sub-analysis of the Scandinavian Simvastatin Survival Study in patients with coronary heart disease and hypercholesterolemia, treatment with 20–40 mg/day of simvastatin reduced the risk of onset or worsening of intermittent claudication by 38% after a median follow-up period of 5.4 years (82). The IDEAL (Incremental Decrease in End Points Through Aggressive Lipid Lowering) study analyzed the effect of one statin at high doses (atorvastatin 80 mg/day) vs. another at usual doses (simvastatin 20–40 mg/day) on the incidence of PAD in 8,888 patients who had suffered a myocardial infarction. After a median follow-up period of 4.8 years, atorvastatin significantly reduced the incidence of PAD compared to simvastatin [Hazard ratio (HR) = 0.70, 95% CI 0.53–0.91; *p* = 0.007] (83). These results point toward the need to use high-potency statins in patients with PAD.

The REACH international registry (REduction of Atherothrombosis for Continued Health) included 5,861 patients with symptomatic PAD, of whom 62% were treated with a statin. At 4-years of follow-up, statin therapy reduced the risk of adverse events of the lower extremities (worsening of claudication symptoms, new episode of CLI, revascularization, or amputation) by 18% (22.0 vs. 26.2%; HR 0.82; 95% CI 0.72–0.92; *p* = 0.0013) (84).

Few clinical trials have examined at the role of statins in intermittent claudication and their results are controversial. Most show that statins improve exercise tolerance by increasing painless walking distance and maximum walking distance. Mohler et al. randomized 354 patients with intermittent claudication into groups which received either a placebo or atorvastatin (10 or 80 mg daily) for 1 year. They found that the group treated with atorvastatin 80 mg/day improved painless walking distance compared to the placebo group and no differences between the latter group and those treated with atorvastatin 10 mg/day (85). A physical activity questionnaire showed improvement in ambulatory capacity in the two atorvastatin treatment groups, but there were no differences between the groups in the maximum distance traveled or on the quality of life questionnaires. In another clinical trial, Mondillo et al. randomized 86 patients with intermittent claudication and hypercholesterolemia into groups which received 40 mg/day of simvastatin or a placebo. After 6 months of treatment, a significant increase of 90 m in painless walking distance was found in the simvastatin-treated group compared to the placebo group, with significant improvements also observed in the total distance traveled (120 m) and in the ABI values at rest and after exercise (86). Aronow et al. investigated the effect of simvastatin treatment 40 mg/day vs. placebo on exercise time on a treadmill at 6 and 12 months in 69 patients >60 years with intermittent claudication and an ABI <0.9. Treatment with simvastatin significantly increased exercise time until intermittent claudication at 6 and 12 months (87). However, not all studies have shown the benefit of statins in increasing painless walking distance. In a small, double-blind clinical trial, 37 patients with hypercholesterolemia and intermittent claudication without previous treatment with statins were randomized to receive atorvastatin 20 mg/day or a placebo for 3 months. At the end of treatment, both groups significantly increased painless walking distance with no differences between them, despite the decrease in LDL-C in the group treated with atorvastatin (88).

The benefits of statins on the functional capacity of patients with PAD appear to be independent of plasma cholesterol levels. In a study conducted in patients with PAD with and without hypercholesterolemia who were treated with simvastatin 40 mg/day for 3 months, an improvement in total pain-free walking distance was observed in both groups (89). Along these lines, another study also found better leg functionality, as assessed *via* various parameters, in patients with and without PAD treated with statins compared to those who did not receive such treatment, regardless of cholesterol levels (90). These results support the idea that all patients with PAD can benefit from statin therapy regardless of their cholesterol levels and suggest that other properties of statins apart from their lipid-lowering action may be responsible for the improvement of intermittent claudication symptoms observed with these drugs. In fact, in a study conducted in 102 patients who undergo endovascular revascularization which compared the effect of simvastatin monotherapy vs. triple therapy with simvastatin, niacin, and ezetimibe, no differences were found between the two groups in the volumes of the superficial femoral artery measured by magnetic resonance imaging despite the fact that triple therapy significantly improved lipid parameters with respect to simvastatin alone (91). On the other hand, it is important to note that various studies suggest that the anti-inflammatory effects of statins are a key element of their benefit in PAD. In patients with severe PAD, statin therapy has been associated with a significant improvement in survival in cases with intense inflammatory activity, as measured by high-sensitivity CRP levels, but did not provide benefits in those with low inflammatory activity, suggesting that statins exert their protective effect through anti-inflammatory mechanisms or by attenuating the deleterious effects of inflammation (92). Vidula et al. analyzed whether statin use was associated with lower cardiovascular and all-cause mortality based on CRP and D-dimer levels in 579 patients with PAD in the Walking and Leg Circulation Study (WALCS) and WALCS II cohorts. After an average follow-up period of 3.7 years, the use of statins was associated with lower cardiovascular and all-cause mortality compared to those who did not use these drugs, without no significant interactions with baseline CRP or D-dimer levels. However, statin therapy was associated with significantly lower mortality only among participants with baseline CRP values above the median, not among those with values below the median (93).

A meta-analysis that involved 43 studies investigated the effect of several drug classes on ambulation capacity and found that lipid-lowering drugs were the most effective, especially statins, with an average increase in maximum walking distance of 160 m whereas other drugs only increased walking distance by 50 m (94). The benefit of statins on exercise tolerance may be greater in patients with moderate-severe arterial stenosis (defined as an ABI <0.7) than in those with mild stenosis, which has been attributed to a possible regression and stability of the atheroma plaque in cases of more advanced atherosclerotic lesions with a higher lipid load on the plaque (95).

Despite these findings, the actual impact of improved exercise tolerance on the quality of life of patients with statin-treated PAD has not been demonstrated (85).

## Effects of Statins on Cardiovascular morbidity and Mortality

Numerous studies strongly support the use of statins in patients with early and advanced stages of PAD to reduce their cardiovascular risk (Tables 1, 2).

**Table 1:** Benefits of statins in patients with early stages of PAD (asymptomatic or symptomatic PAD).

**References**	**Year of publication**.	**N**	**Study**	**All-cause mortality HR; 95% CI**	MACE^*^ HR; 95% CI	**Cardiovascular mortality HR; 95% CI**	MALE^*^ HR; 95% CI	**Amputation HR; 95% CI**	**Pain-free walking distance**
Peters et al. (96)	2020	22,208	P	0.80; 0.70–0.92 *p* < 0.05	0.80; 0.70–0.92 *p* < 0.05	NA	NA	NA	NA
Kumakura et al. (21)	2019	932	P	0.46; 0.35–0.59 *p* < 0.001	NA	NA	NA	NA	NA
Arya et al. (34)	2018	155,647	R	0.74; 0.70–0.77 *p* < 0.05	NA	NA	NA	0.67; 0.61–0.74 *p* < 0.05	NA
Tern et al. (97)	2018	678	R	0.56; 0.37–0.85 *p* < 0.01	NA	NA	NA	NA	NA
Arao et al. (95)	2017	75	P	NE	NA	NA	NA	NA	NS
Foley et al. (98)	2017	909	R	0.52; 0.33–0.8 *p* = 0.004	0.58; 0.37–0.92 *p* = 0.02	NA	NA	NA	NA
Thatipelli et al. (99)	2017	395	R	0.28; 0.20–0.39 *p* < 0.001	NA	NA	NA	NA	NA
Hsu et al. (100)	2017	64,902	R	0.73; 0.69–0.77 *p* < 0.05	NA	0.78; 0.69–0.87 *p* < 0.05	NA	0.75;0.62–0.90 *p* < 0.05	NA
Ramos et al. (101)	2016	5,480	R	0.81; 0.68–0.97 *p* < 0.05	0.80; 0.66–0.97 *p* < 0.05	NA	NA	NA	NA
Vrsalović et al. (185)	2015	319	R	NA	NA	NA	NA	NA	NA
Kumbhani et al. (84)	2014	5,361	R	0.83; 0.72–0.96 *p* = 0.014	0.83; 0.73–0.96 *p* = 0.01	0.84; 0.70–1.00 *p* = 0.05	0.82; 0.72–0.92 *p* = 0.0013	0.64; 0.48–0.86 *p* = 0.002	NA
Vidula et al. (93)	2010	679	P	0.51; 0.30–0.86 *p* = 0.012	NA	0.36;0.14–0.89 *p* = 0.027	NA	NA	NA
De Liefde et al. (102)	2008	2,109	P	0.59; 0.44 −0.79 *p* < 0.05	0.59; 0.43–0.81 *p* < 0.05	NA	NA	NA	NA
HPSCG (103)	2007	6,748	RCT	NS	0.78; 0.71–0.85 *p* < 0.0001	NS	NS	NS	NA
Feringa et al. (104)	2007	1,374	P	0.71; 0.62–0.80 *p* < 0.05	NA	0.76; 0.67–0.86 *p* < 0.05	NA	NA	NA
Pasqualini et al. (105)	2007	357	P	0.46; 0.21–0.96 *p* = 0.038	NA	0.43; 0.16–1.12 *p* = 0.08	NA	NA	NA
Schillinger et al. (92)	2004	515	P	0.52; 030–0.91 *p* = 0.022	0.48; 0.29–0.79 *p* = 0.004	NA	NA	NA	NA
McDermott et al. (90)	2003	392	P	NA	NA	NA	NA	NA	1276 vs. 1218 (foots) *p* = 0.045
Mohler et al. (85)	2003	364	RCT	NA	NA	NA	3 vs. 9% *p* < 0.003	NA	63 vs. 38% (s) *p* < 0.025
Aronow et al. (87)	2003	660	RCT	NA	NA	NA	NA	NA	Increase 42% (s) *p* < 0.0001
Mondillo et al. (86)	2003	86	RCT	NA	NA	NA	NA	NA	Increase 90 (m) 95% CI 64 to 116 *p* < 0.005

*N, number of patients; HR, hazard ratio; CI, 95% confidence interval; NA, non-available; NS, non-significant*.

*PAD, peripheral arterial disease; MACE, major adverse cardiovascular event (cardiovascular death, myocardial infarction, stroke); MALE, major adverse limb event (death, clinical worsening, need for revascularization or amputation); HPSCG, Heart Protection Study Collaborative Group; RCT, randomized clinical trial; R, retrospective observational study; P, prospective observational study*.

* *Heterogeneous definitions were used between the different studies. For more information, see references*.

**Table 2:** Benefits of statins in patients with advanced stages of PAD (CLI or undergoing revascularization).

**References**	**Year of Public**.	**N**	**Study**	All causes mortality^*^ HR; 95%CI	MACE^*^ HR; 95%CI	MALE^*^ HR; 95%CI	Patency^*^ HR; 95%CI	**Revascularization**	**Amputation HR; 95%CI**
Jung et al. (106)	2021	952	R	NA	NA	NA	75.2 vs. 88.8% *p* = 0.004	NA	NA
Peters et al. (96)	2020	22,208	P	0.75; 0.68–0.84 *p* < 0.05	NS	NA	NA	NE	0.7; 0.58–0.93 *p* < 0.05
Moore et al. (107)	2020	105,628	R	91 vs. 87% *p* < 0.001	NA	NA	NA	NA	NA
Parmar et al. (108)	2019	488	R	88.8 vs. 77.5% *p* < 0.0001	NA	90.7 vs. 79.3%*p* = 0.002	NA	NA	NA
Khan et al. (109)	2019	1204	R	0.7; 0.6–0.9 *p* = 0.001	0.7; 0.6–0.8 *p* < 0.001	NA	NA	NA	NA
De Martino et al. (110)	2016	14,396	R	NA	NS	NA	NA	NA	NA
DeCarlo et al. (111)	2017	811	P0	0.46;0.29–0.73 *p* < 0.05	NA	NA	NA	NA	NA
Matsubara et al. (112)	2017	114	R	NA	0.38; 0.16–0.78 *p* < 0.05	NA	NA	NA	NA
Siracuse et al. (113)	2017	1014	P	NS	NA	0.18; 0.07–0.47 *p* < 0.001	NA	NS	NS
O'Donnell et al. (114)	2017	931	R	0.7; 0.60–0.90 *p* < 0.01	NA	0.71; 0.51–0.97 *p* < 0.001	NA	NA	NA
Stavroulakis et al. (115)	2017	816	R	0.40; 0.24–0.66 *p* < 0.001	0.41; 0.23–0.69 *p* = 0.001	NA	NA	NA	NA
Klingelhoefer et al. (116)	2016	244	R	NA	NA	NA	NS	NA	NS
Lee et al. (117)	2016	342	R	2 vs. 7 % *p* = 0.007	4 vs. 10% *p* = 0.002	NA	NA	NA	NA
Kim et al. (118)	2016	135	R	NA	NA	NA	72.5 vs. 50.5% *p* = 0.01	35.2 vs. 54.5% *p* = 0.03	NA
Jones et al. (119)	2015	908	P	NA	0.66; 0.48–0.88 *p* < 0.001	NA	NA	NA	NA
Iida et al. (120)	2015	314	P	NA	NA	0.28;0.10–0.78 *p* = 0.02	NA	NA	NA
Suckow et al. (121)	2015	2,067	P	0.7; 0.6–0.9 *p* < 0.001	38 vs. 22% *p* < 0.001	NA	NS	50 vs. 38% *p* < 0.001	NS
Spiliopoulos et al. (122)	2015	214	R	0.5; 0.31–0.98 *p* < 0.04	NA	NA	NA	NA	NA
Westin et al. (123)	2014	380	R	0.49; 0.24–0.97 *p* < 0.05	0.53; 0.28–0.99 *p* < 0.05	0.53; 0.35–0.98 *p* < 0.05	0.45; 0.22–0.89 *p* < 0.05	NA	NS
Siracuse et al. (124)	2014	221	R	0.5; 0.28–1.0 *p* < .001	NA	NA	0.39; 0.23–0.67 *p* < 0.05	NA	NA
Baril et al. (125)	2013	5,706	R	0.80; 0.71–0.91 *p* < 0.0007	NA	NS	NA	NA	NS
Brunner et al. (91)	2013	102	RCT	NS	NS	NS	NA	NS	NA
Tomoi et al. (126)	2013	812	R	64.5 vs. 45.9 %*p* = 0.004	NA	NS	NA	43.6 vs. 54.6% *p* = 0.03	64.1 vs. 43.0% *p* = 0.003
Saqib et al. (127)	2013	210	R	NA	NA	NA	NS	NA	NA
Vogel et al. (128)	2013	22,954	R	NA	NA	0.82; 0.78–0.86 *p* < 0.0001	NA	NA	0.80; 0.75–0.86 *p* < 0.0001
Todoran et al. (129)	2012	136	P	0.16; 0.03–0.90 *p* = 0.034	NA	NA	NA	NA	0.11; 0.2–0.6 *p* = 0.01
Aiello et al. (130)	12	646	R	7 vs. 62% *p* = 0.038	NA	NA	66 vs. 51% *p* = 0.001	NA	NA
Siracuse et al. (131)	2012	218	R	NA	NA	NA	0.6; 0.35–0.97 *p* < 0.05	NA	NA
Scali et al. (132)	2011	116	R	NA	NA	NA	0.49; 0.25–0.96 *p* = 0.038	NA	NA
Isma et al. (133)	2008	259	P	75 vs. 52% *p* = 0.0012	NA	NA	NA	NA	NA
Schanzer et al. (134)	2008	1,404	P	0.71; 0.52–0.98 *p* = 0.03	NS	NA	NS	NA	NA
Van Gestel et al. (135)	2008	3,371	R	0.67; 0.52–0.86 *p* < 0.05	NA	NA	NA	NA	NA
Ward et al. (136)	2005	446	P	0.52; 0.32–0.84 *p* < 0.05	0.36; 0.14–0.93 *p* = 0.03	NS	NA	NA	NA
Henke et al. (137)	2004	293	R	NS	NA	NA	3.7; 2.1–6.4 *p* < 0.001	NA	0.34; 0.77–6.15 *p* = 0.01
Abbruzzese et al. (138)	2004	172	R	NS	NA	NA	97 vs. 87% *p* < 0.02	NA	NA

*N, number of patients; HR, hazard ratio; CI, 95% confidence interval; NA, non-available; NS, non-significant*.

*PAD, peripheral arterial disease; CLI, critical limb ischemia; MACE, major adverse cardiovascular event (cardiovascular death, myocardial infarction, stroke); MALE, major adverse limb event (death, clinical worsening, need for revascularization or amputation); HPSCG, Heart Protection Study Collaborative Group; RCT, randomized clinical trial; R, retrospective observational study; P, prospective observational study*.

* *Heterogeneous definitions between the different studies. For more information, see references*.

The Heart Protection Study (HPS) was the study that included the largest number of patients with PAD. In this clinical trial, 20,536 patients at high cardiovascular risk (6,748 with PAD) were randomized to receive simvastatin 40 mg/day or a placebo for an average of 5 years. In the group of patients with PAD, treatment with simvastatin led to a decrease of 22% (95% CI 15–29; *p* < 0.0001) in the risk of suffering a major vascular event (myocardial infarction, coronary death, stroke, or revascularization), regardless of baseline LDL-C levels (103). This study again shows that the benefit of statin therapy in patients with PAD is independent of cholesterol levels and supports its routine use in this disease.

In the REACH registry, statin treatment was associated with a 17% decrease in the combined outcome variable of cardiovascular death, non-fatal infarction, and non-fatal stroke (HR 0.83; 95% CI, 0.73–0.96; *p* = 0.01) (84). In a Cochrane review, the effects of treatment with various lipid-lowering drugs on all-cause mortality, cardiovascular events, and local disease progression in patients with PAD was analyzed (139). Eighteen randomized clinical trials were included, of which only three used statins, with a total of 10,049 participants. The overall results of all trials included showed that lipid-lowering therapy had no effect on overall mortality (six studies) or on total fatal and non-fatal cardiovascular events (eight studies). A sub-analysis of the two studies investigating the effects of statins on total cardiovascular events–the HPS with simvastatin 40 mg/day (103) and the Mohler study with atorvastatin 10 and 80 mg/day (85)–showed a significantly lower risk of cardiovascular events in subjects treated with statins compared to those treated with a placebo (OR 0.74; 95% CI 0.67–0.82; *p* < 0.00001). Another review of studies published after 2000 on the effect of statins on cardiovascular events and mortality confirmed that there is strong evidence supporting the benefit of statins on cardiovascular morbidity and mortality in patients with PAD (140).

In a Spanish observational study conducted in primary care departments on asymptomatic patients with an ABI <0.95 and without cardiovascular disease, the effect of statins on the incidence of major adverse cardiovascular events (infarction, cardiac revascularization, and ischemic stroke) and all-cause mortality was analyzed (101). Both the incidence of cardiovascular events and all-cause mortality were lower in patients treated with statins. This study shows that statins are useful for decreasing mortality and cardiovascular events, even in patients with low-risk PAD and that all patients with PAD should be treated with statins regardless of their symptoms or cardiovascular risk.

Other studies also show reduced mortality in patients with PAD who receive statins. Feringa et al. conducted a prospective observational study in 1,374 patients with PAD (defined as an ABI <0.9) to determine whether higher doses of statins and lower LDL-C levels are independently associated with a better clinical course of the disease. The mean follow-up was 6.4 years and the primary objective was cardiac and all-cause mortality. The multivariate analysis found that both higher doses of statins and lower LDL-C levels were independently associated with lower cardiac and total mortality (104). In addition, statins appear to be the drug class that contributes most to the reduction in mortality in patients with PAD. A prospective study of 2,420 patients with an ABI ≤ 0.9 followed for 8 years analyzed the effect of various cardioprotective drugs on all-cause mortality and found that, in addition to beta-blockers, aspirin, and angiotensin-converting-enzyme inhibitors (ACEI), statin use was associated with a reduction in mortality risk (HR 0.46; 95% CI 0.36–0.58); they were the drugs that provided the greatest benefit (141).

Elderly patients with PAD also seem to benefit from reduced cardiovascular risk with statins. A prospective observational study in a cohort of 660 elderly patients (mean age 80 years) with symptomatic PAD and serum LDL-C levels >125 mg/dL found that the incidence of new coronary events was 48% if they received statins and 73% if they did not take lipid-lowering drugs (142).

From the above studies, it can be deduced that all patients with PAD, regardless of their LDL-C levels, should be treated with statins in order to reduce cardiovascular morbidity and mortality.

## Effects of Statins in Patients Undergoing Revascularization Procedures

Revascularization should be considered in patients with symptomatic PAD that decreases their functional capacity. Advances in endovascular revascularization techniques have led to these procedures being used more and more frequently for the treatment of PAD. Several studies have been conducted in patients undergoing endovascular or surgical revascularization treatment to analyze the effect of statin treatment on the viability of the operated extremities as well as perioperative morbidity and mortality and survival. Although not all studies have shown favorable results, there are sufficient data to recommend its use in this population.

In a registry of 1,357 patients with PAD and stable claudication undergoing percutaneous revascularization at the aortoiliac or femoropopliteal level, the effect of aspirin and statin intake on cardiovascular events and complications in the legs at 6 months was analyzed. The rate of peripheral adverse events, including the need to repeat endovascular intervention or require amputation or revascularization surgery to save the limb, was lower in those receiving the drugs (odds ratio, 0.45; 95% CI, 0.29–0.71) (143). Abbruzzese et al. conducted a retrospective study of 172 patients with PAD who underwent infrainguinal bypass to study the influence of statin therapy on the permeability of major saphenous vein grafts and found that those taking statins had higher rates of graft permeability at 2 years (138). In another retrospective study, Parmar et al. evaluated the effect of statin use on limb conservation and survival in 488 patients with PAD undergoing surgical or endovascular revascularization treatment. Patients treated with statins had a 69% reduction in the rate of amputations (HR 0.31; 95% CI: 0.14–0.68), in addition to an improvement in limb recovery at 30 days and at 1 and 5 years after adjusting for age, race, sex, type of intervention (surgical or endovascular), indication for the intervention, diabetes, hypertension, coronary heart disease, end-stage renal failure, tobacco use, and antiplatelet therapy (108). However, these very favorable data have not been corroborated in other works. In one study, the use of statins was associated with better lesion permeability in patients undergoing infrapopliteal angioplasty and greater amputation-free survival, but did not decrease the rate of amputations (123). Another very recent study in patients with bypass observed that statin therapy was associated with long-term graft permeability on the univariate analysis, but was no longer an independent factor on the multivariate analysis (106).

Study results are also mixed in patients treated with stents. In patients treated with these devices for femoropopliteal lesions, statin therapy decreased the rate of restenosis at 1 and 2 years (118). However, another study did not demonstrate a benefit of statins in improving the permeability of stents implanted in popliteal or femoral arteries, although it found a tendency for improvement in less severe lesions (144).

Regarding the role of statins in the perioperative course of patients treated with revascularization procedures, studies have also shown heterogeneous results. Antoniou et al. published a meta-analysis that included 24 studies (20 observational and 4 randomized clinical trials) analyzing the effect of statins on mortality and cardiovascular events in the perioperative period in patients undergoing non-cardiac surgical or endovascular revascularization processes. The use of statins was associated with a decreased risk of all-cause mortality (OR 0.54; 95% CI 0.38-0.78), myocardial infarction (OR 0.62; 95% CI 0.45–0.87), stroke (OR 0.51; 95% CI 0.39–0.67) and the combined outcome of myocardial infarction, stroke, or death (OR 0.45; 95% CI 0.29–0.70) (145). Another study retrospectively examined the effect of preoperative statin therapy on perioperative cardiac and vascular outcomes and long-term survival in 446 patients undergoing infrainguinal bypass surgery. Statin therapy was associated with fewer cardiovascular complications, a shorter mean length of hospital stay, and higher survival during the mean follow-up period of 5.5 years, but perioperative mortality did not decrease (136); the same was found by the aforementioned Abbruzzese study (138). Similarly, another retrospective study based on the Vascular Quality Initiative database suggests that statins may not be associated with perioperative improvement when other risk factors are considered as covariates. This study, which included patients undergoing supra- and infrainguinal bypass surgery or infrarenal abdominal aortic aneurysm repair, analyzed the association between preoperative statin use and postoperative in-hospital myocardial infarctions and combined myocardial infarction/death. After multivariate adjustment, preoperative statin use was not associated with a reduction in the rate of infarction/death, while estimation of previous cardiac risk and intraoperative blood loss did (110).

Numerous studies support the use of statins in patients with PAD after revascularization to decrease mortality (Table 2). A recent retrospective study analyzed the effect of initiating statin therapy on all-cause mortality, cardiovascular events, and major amputations in 10,922 patients with chronic limb-threatening ischemia and intermittent claudication undergoing revascularization treatment without previous statin treatment. After a 5-year follow-up period, statin initiation was associated with lower mortality in both groups, a lower risk of increased amputation in patients with chronic limb-threatening ischemia, and a lower risk of cardiovascular events in patients with intermittent claudication (96). Other studies also show lower mortality at 1 (125, 134), 5 (121), and 10 years (135) in patients with PAD treated with statins when undergoing surgical revascularization treatment.

There are more data confirming lower cardiovascular morbidity and mortality and better progression in the lower extremities in patients treated with statins after endovascular or surgical revascularization (84, 109, 112, 113, 116, 119, 124, 128, 131, 132, 137, 146, 147). A recent study examined changes in medication prescribing and adverse events in a Danish national cohort of patients with symptomatic PAD after undergoing a revascularization procedure between 2000 and 2016. An increase in antiplatelet agent and statin prescribing during that period was associated with a decrease in major cardiovascular events, myocardial infarction, and cardiovascular and all-cause mortality, but not in amputations (148). These results reinforce the indication of statins in patients undergoing revascularization of the lower extremities. Despite this, there is evidence that these patients are subtreated (128, 137, 149). For example, one study which included patients who had had an infrainguinal bypass between 1997 and 2002 found that only 56% of them were treated with statins (137). In addition, it seems that these patients should not only be treated with statins, but should also achieve low LDL-C levels. Tomoi et al. investigated the association between LDL-C levels and cardiovascular death in 935 patients with PAD undergoing endovascular revascularization treatment. LDL-C levels above 100 mg/dL at 3–6 months of the intervention were independently associated with an increased risk of cardiovascular death at 5 years, even in patients receiving statins (150).

In summary, data from studies in patients with PAD undergoing revascularization procedures support the use of statins to reduce cardiovascular events and mortality, but show discordant results in regard to the perioperative course and permeability of revascularization or the amputation rate.

## Effects of Statins on Severe Limb Ischemia and Amputations

CLI is associated with a significantly higher need for amputation and mortality than in patients with intermittent claudication (151). The benefits of statins have been confirmed in numerous studies of patients with severe PAD or CLI; those treated with statins had lower mortality and major cardiovascular event rate as well as better outcomes for survival without amputation and limb conservation (34, 84, 100, 111, 115, 120, 122, 123, 129, 130, 133, 137, 138, 140, 152, 153). In some studies, this benefit appears to be related to the potency of statins and has only been observed in moderate- and high-intensity statins (111).

The most controversial aspect in patients with severe ischemia is the role of statins in reducing amputations. An observational study from Taiwan's national diabetes database investigated whether statin use was associated with a lower risk of amputation in patients with type 2 diabetes and PAD. Compared to those who did not use statins, those who took these drugs had a lower risk of limb amputation (adjusted HR 0.75; 95% CI 0.62–0.90) (100). Another retrospective study conducted in a cohort of 83,593 patients with type 1 and type 2 diabetes compared the incidence of amputations in those treated with statins to those with lipid-lowering drugs without statins or those without lipid-lowering drugs and found that, compared to patients who did not receive lipid-lowering drugs, those treated with statins had a 35% reduction in the risk of amputation (HR 0.65; 95% CI 0.42–0.99) and 43% of the amputation or death combination (HR 0.57; 95% CI 0.54–0.60), (154). This protective effect was not observed in diabetic patients treated with lipid-lowering drugs other than statins, whose rate of amputations was similar to that of those who did not receive lipid-lowering drugs (HR 0.95; 95% CI: 0.35–2.60). This finding again points to the fact that the benefit of statins is not only due to their effect on lipids, but to their pleiotropic actions at other levels. A recent systematic review and meta-analysis investigated the impact of statins on major adverse limb events (graft amputation and occlusion/revascularization) and cardiovascular morbidity and mortality in patients with PAD. Fifty-one studies were included (2 randomized controlled trials, 20 prospective studies, and 29 retrospective studies) involving 138,060 patients, of which 35.1% were treated with statins. In addition to demonstrating a clear reduction in the risk of death and cardiovascular events, statins reduced the incidence of major adverse limb events by 30% (pooled HR 0.702; 95% CI 0.605–0.815). When a specific sub-analysis on amputations was performed, statins were found to reduce the amputations rate by 35% (pooled HR 0.654; 95% CI 0.522–0.819) (152). Another systematic review and meta-analysis of studies conducted in patients with CLI which included 19 studies (4 prospective clinical trials and 15 retrospective and non-randomized trials) with 26,985 patients, of which almost 50% were treated with statins, concluded that those treated with these drugs were 25% less likely to have amputation (HR 0.75; 95% CI: 0.59–0.95) (153).

However, these positive results in terms of reduction of amputations have not been confirmed in other studies (115, 121, 123, 134, 140, 148). It has been suggested that the high rate of arteriosclerosis in CLI may limit the impact of statins observed in patients with coronary heart disease (115). HPS data show that simvastatin treatment significantly reduced the rate of a first peripheral vascular event (endarterectomy or carotid angioplasty, other arterial grafts, or angioplasty and amputation), but there was no difference in the incidence of amputations between the simvastatin-treated group and the placebo-treated group (103). The CRITISCH (First-Line Treatments in Patients With Critical Limb Ischemia) registry, in a prospective study of 1,200 patients with CLI, showed that the use of statins increased time until the need for major amputation and reduced mortality and major cardiovascular events, but not the rate of amputations (115). Another retrospective Japanese study, which examined the efficacy of statin therapy after endovascular treatment in isolated below-knee lesions in 812 patients with CLI found that, after adjusting for several variables, there were no significant differences between the statin-treated and non-statin-treated groups in overall survival, amputation-free survival, cardiovascular death, limb preservation, need for repeated revascularization, and major adverse limb events (repeated revascularization or major amputation) (126).

In summary, although the benefit of statins on the reduction of amputations seems controversial, there is sufficient evidence for their indication in patients with CLI due to the decrease in cardiovascular morbidity and mortality they provide.

## Recommendations on the Use of Statins in Peripheral Arterial Disease According to the Different Guidelines

The recommendations of PAD management guidelines are aimed at improving patients' symptoms and quality of life, preserving lower extremities, and reducing mortality and the risk of developing cardiovascular events. To this end, in addition to lifestyle modifications and tobacco cessation, it is recommended that patients with PAD receive treatment to control cardiovascular risk factors, including antihypertensive drugs, antiplatelet drugs, and lipid-lowering drugs. Statins are first-line lipid-lowering drugs in the treatment of patients with PAD and the main reason for their indication is the reduction of cardiovascular morbidity and mortality they provide.

Nowadays, all guidelines aimed at reducing the risk of developing atherosclerotic disease in any vascular territory advise the use of statins, both in primary and secondary prevention. The 2018 American College of Cardiology (ACC) and American Heart Association (AHA) guidelines on cholesterol management recommend treatment with a high-intensity statin treatment or at the maximum tolerated dose in all patients with clinical atherosclerotic vascular disease (coronary, cerebrovascular, or peripheral) (28). However, in the case of PAD, many of the recommendations in the guidelines are based on extrapolations from studies conducted in coronary patients, as there are few data derived from studies conducted specifically in patients with PAD. The most recent PAD management guidelines recommend statin therapy in all patients with class I and level of evidence A (1, 9).

The 2018 ACC/AHA guidelines for the treatment of hypercholesterolemia suggest reducing LDL-C levels with statins of greater or lesser intensity depending on the patient's risk (28). Although these guidelines do not establish LDL-C targets, they recommend that patients with atherosclerotic cardiovascular disease should achieve a ≥50% reduction in LDL-C levels. For patients with PAD, they recommend the use of a high-intensity statin (one that achieves a decrease in LDL-C ≥50%) if they are ≤ 75 years of age (recommendation IA). In the event that after treatment with the maximum tolerated statin dose LDL-C levels remains ≥70 mg/dL, it may be advisable to add ezetimibe. For patients >75 years of age, a high- or moderate-intensity statin should be considered after weighing the possible reduction of cardiovascular risk, adverse effects, drug interactions, and patient frailty and preferences. In patients with PAD in whom high-intensity statins are contraindicated or who have adverse effects with them, treatment with a moderate-intensity statin should be initiated to achieve a reduction in LDL-C from 30 to 49%. In patients with very high-risk PAD, which would be those with intermittent claudication and ABI <0.85, or with a history of revascularization or amputation, lipid-lowering therapy should include the maximum tolerated doses of statins and ezetimibe before considering treatment with proprotein convertase subtilisin/kexin type 9 inhibitors (PCSK9-I).

The 2016 ACC/AHA guidelines for the treatment of patients with PAD recommend statin therapy in all cases, but do not set LDL-C targets (9). In contrast, the European guidelines, in addition to indicating statins to all patients, propose a target LDL-C <70 mg/dL or a reduction ≥50% if baseline levels are 70–135 mg/dL and propose combination treatment with ezetimibe in select patients (1). The latest European guidelines for the treatment of dyslipidemia set even lower LDL-C targets for patients with PAD (<55 mg/dL and with a reduction ≥50% from baseline) and considers them to be a very high cardiovascular risk group. To achieve these LDL-C targets, the use of a high-intensity statins at the maximum tolerated dose is recommended, adding ezetimibe or PCSK9-I if necessary (29). It is important to achieve these objectives early because it has been demonstrated in patients with PAD undergoing endovascular revascularization that achieving an LDL-C <70 mg/dL in the short term (mean 4.8 months) reduces mortality and cardiovascular events (117).

## High- or Low-Intensity Statins in Patients With Peripheral Artery Disease?

The use of high-intensity statins recommended by the guidelines reduces local and cardiovascular complications in patients with PAD, with an inverse relationship between the intensity of the statins used and the risk of death or amputation. For patients not treated with statins, those who received high-intensity statin treatment at the time of PAD diagnosis showed a nearly 30% reduction in the risk of death and a 30–40% decrease in the risk of major amputation, while those who had mild- or moderate-intensity statin treatment had a lower degree of benefit (34). A *post hoc* analysis of the IDEAL study that analyzed the effect of atorvastatin 80 mg/day vs. simvastatin 20–40 mg/day on the risk of cardiovascular events in patients with PAD observed a significant decrease in cardiovascular and coronary events and coronary artery bypass in those treated with atorvastatin (83). In a recent observational study, 155,647 patients with PAD from the U.S. Veterans Health Administration database were classified according to treatment with high-intensity or low-intensity to moderate-intensity statins, using those who did not receive statins (28% of patients) as a control group. After an average follow-up period of nearly 6 years, it was found that, for patients not receiving statins, both high-intensity and moderate-intensity statins reduced mortality (30 and 20%, respectively) and amputations (40 and 20%, respectively). However, patients treated with high-intensity statins had a 15% lower risk of mortality and 22% lower risk of amputation than those who received low- or moderate-intensity statins (34).

In patients with symptomatic PAD undergoing arteriography and/or endovascular revascularization, those treated with high-intensity statins (atorvastatin 40–80 mg or rosuvastatin 20–40 mg) had a higher survival (HR for mortality: 0.52; 95% CI 0.33–0.81; *p* = 0.004) and suffered fewer major cardiovascular events (HR 0.58; 95% CI 0.37–0.92, *p* = 0.02) than those treated with low-to moderate-intensity statins, although LDL-C levels in both groups were similar (98). These results suggest that the beneficial effect of high doses of statins may be derived from their pleiotropic actions.

All these studies underline the importance of initiating high-intensity statins in patients with PAD from the moment of diagnosis and in the event these are not tolerated, at least one low- or moderate-intensity statin. However, when prescribing high-intensity statins it is important to note that although this treatment provides greater benefits than low or moderate doses, its use is also associated with an increase in adverse effects. Although these drugs are very safe, care must be taken regarding possible interactions with other drugs and the patient must be monitored for the onset of muscular symptoms, diabetes (especially in patients with metabolic syndrome or pre-diabetes), or alterations in the liver profile. Other purported adverse effects of statins such as cognitive impairment, a significant decline in kidney function, or increased risk of cataracts or hemorrhagic stroke have not been confirmed (155).

## Statin Prescription Rate in Patients With Peripheral Arterial Disease

The higher the cardiovascular risk, the greater the benefit of statins. Given the high risk of PAD, these patients may have a marked decrease in their risk when treated with statins. There is strong evidence that the follow-up on statin therapy recommended by the guidelines improves survival and decreases lower limb complications in patients with PAD (114). However, despite the guidelines' recommendations, the prescribing of statins in general and of high-intensity statins in particular in patients with PAD is low compared to that of patients with coronary or carotid disease (16, 19, 27, 33, 34, 156–161). The reasons for this low prescribing rate are diverse. One of the most important reasons is likely the lack of specifically designed studies in patients with PAD that support the use of statins in this population. In addition, unlike patients with coronary heart disease, patients with PAD are not recognized as a high cardiovascular risk population by some medical specialties and accordingly, the statin prescribing rate differs among them (162). The increased awareness of coronary patients' high cardiovascular risk and the need to decrease their LDL-C levels explains the increased use of statins and other cardioprotective drugs in patients with PAD and coronary heart disease (33, 34, 84, 156, 163–165).

According to data from the National Health and Nutrition Examination Survey (NHANES) from 1999 to 2004, statins were used in only 30% of patients with PAD defined by an ABI ≤ 0.9 (152). Subsequent studies have shown increased use of these drugs in PAD (33, 159, 160, 166–169). A study conducted in patients with PAD attended to in a tertiary hospital in Switzerland between 2010 and 2017 analyzed the prescribing of statins and the degree of LDL-C control. It observed a significant increase in the prescribing of lipid-lowering drugs (59% in 2010 to 81% in 2017) and a parallel decrease in mean LDL-C values (110 vs. 80 mg/dL, *p* < 0.0001 for the trend) (168). In a review and meta-analysis that investigated the rate of prescribing of cardioprotective drugs in patients with PAD and included 86 studies published since 2000, it was observed that although the prescribing of these drugs has improved over the years, their use remains low. The use of antiplatelet agents, statins, and ACEI/angiotensin II receptor blockers was 75, 56, and 53%, respectively, with an increase in the prescribing rate of statins according to the year of publication of the studies (+2.0% per year, *p* < 0.001) that was not observed in antiplatelet agents or ACEI/angiotensin II receptor blockers (160).

However, the prescribing of statins in patients with PAD remains low and the use of the doses recommended by the guidelines continues to be insufficient, which means that the desired LDL-C targets are not met in a considerable number of patients (163, 168, 170, 171). Recently, the HCHS/SOL study, conducted in the Latino population established in the United States of America, has shown that only one in four subjects with PAD receives lipid-lowering treatment and one in three receives antiplatelet agents (163). Another recent study from the Vascular and Endovascular Research Network looked at the control of cardiovascular risk factors in 440 patients with PAD from 10 vascular centers in the United Kingdom. Although about 80% of patients were treated with statins, only 11% were treated with a high-intensity statin (atorvastatin 80 mg or similar), as recommended by the guidelines, and the mean LDL-C values in the sample were 104 mg/dL, well above the established targets (171). The PORTRAIT (Patient-Centered Outcomes Related to Treatment Practices in Peripheral Arterial Disease: Investigating Trajectories) registry prospectively analyzed the rate of statin intensification (onset or increase in intensity) in a cohort of patients with PAD evaluated in specialized centers in several countries for the onset or worsening of symptoms. Although the majority of patients received statin therapy according to the guidelines' recommendations, only 31% of those who did not had their treatment intensified. In addition to observing great variability in prescribing between different countries and medical specialties, elderly patients and patients with typical symptoms were less likely and more likely, respectively, to have their treatment intensified (170). In addition to age (19), it appears that sex also influences the prescribing of statins in patients with PAD, such that men were more likely to receive statins than women (19, 172–174), although this does not translate into better LDL-C targets (172).

In addition to statin therapy, it should be ensured that patients with PAD also follow the rest of the pharmacological recommendations indicated by the guidelines (treatment with antiplatelet agents, ACEI, and tobacco cessation), given that patients who meet these four recommendations had a reduction in mortality and major cardiovascular and limb events compared to patients who do not (175). In another study, patients with PAD included in a guidelines-based risk reduction education program, which promoted overall control of cardiovascular risk factors and the use of drugs with demonstrated cardiovascular benefit, including statins, had a reduction in cardiovascular and lower extremity events at seven years of follow-up (176). However, the prescribing of these cardioprotective drugs remains insufficient and one-third of patients undergoing surgical revascularization do not receive them (177).

It is necessary to raise awareness among physicians about following the guidelines' recommendations and taking advantage of any contact between patients and health professionals, especially hospital admission for any reason, to review the cardioprotective treatment of patients with PAD and initiate statin therapy in patients who do not receive them (178). In this sense, the multidisciplinary management of patients undergoing revascularization seems to lead to favorable results and is associated with a high rate of statin prescribing at hospital discharge (179).

In addition to an adequate and early prescription of statins, another important aspect is to achieve good treatment adherence. Up to 12.5% of patients with severe PAD abandoned statin therapy in the first year after diagnosis of the disease (169). In patients with PAD ≥ 65 years who were followed-up on for 5 years, 37.5% abandoned treatment during this time for at least 6 months, 18% during the first year (180). Abandonment of statin therapy, regardless of the cause (lack of adherence or intolerance) leads to decreased survival (107). New avenues have been explored to promote greater statin prescribing and adherence in patients with PAD. It has been shown that providing counseling via telephone to these patients so that they may ask their physicians for more intensive lipid-lowering treatment can improve LDL-C control (181).

## Issues to be Resolved

Although statins are the first-line treatment in patients with PAD, there are currently other potent lipid-lowering drugs that have shown cardiovascular benefits in high-risk patients. Combined treatment of statins and PCSK9-I has been shown to decrease the risk of developing PAD in patients with coronary heart disease (182) and cardiovascular morbidity and mortality in patients with PAD (183). It is necessary to analyze the role of combined statin therapy with the new lipid-lowering drugs (bempedoic acid, inclisiran) on cardiovascular events and complications of the lower extremities in patients with PAD and to conduct comparative studies that clarify whether there are differences between them. We do not have studies that analyze whether achieving strict LDL-C targets, as proposed by the European guidelines (29), provides better prognostic results than treatment with high-intensity statins without set objectives, as indicated by the US guidelines (28). Studies should also be conducted to clarify the effect of statin intensity on the progression of PAD and on amputations and quality of life. A recent study has shown that intramuscular administration of pitavastatin-incorporated nanoparticles may improve lower extremity functionality in patients with chronic limb-threatening ischemia (184). More studies are needed to confirm the benefit and safety of this new treatment. However, the main challenge is to ensure that all medical personnel who have contact with patients with PAD are aware of the high cardiovascular risk associated with this disease and the need to control all patients' risk factors, emphasizing the universal prescription of statins in this population.

## Author Contributions

SJ-C: methodology, writing-original draft preparation, writing-review and editing, and supervision. ML-C, LC-P, JS-C, and MB-L: data curation, writing-original draft preparation, and writing-review and editing. RG-H: writing-original draft preparation, writing-review and editing, and supervision. All authors have read and agreed to the published version of the manuscript.

## Conflict of Interest

The authors declare that the research was conducted in the absence of any commercial or financial relationships that could be construed as a potential conflict of interest.

## Publisher's Note

All claims expressed in this article are solely those of the authors and do not necessarily represent those of their affiliated organizations, or those of the publisher, the editors and the reviewers. Any product that may be evaluated in this article, or claim that may be made by its manufacturer, is not guaranteed or endorsed by the publisher.
